# Current methodologies utilized in the conduct of randomized clinical trials

**DOI:** 10.4155/fsoa-2018-0043

**Published:** 2018-06-26

**Authors:** Marina A Malikova

**Affiliations:** 1 Boston University, Boston Medical Center, Department of Surgery, 88 East Newton Street, D507a Collamore Building, Boston, MA 02118, USA

**Keywords:** clinical trial design, clinical trial methodologies, randomization techniques

Clinical research studies, based on the study design and scientific approach, can be divided into clinical trials and observational studies. In clinical trials, investigators evaluate the effect of intervention (i.e., drug, device, biologic or combination product) on the group of research subjects and assess outcomes prospectively. In observational studies, researchers observe and collect data without intervening in the course of events. Observational studies can be prospective by design, when data are collected from the study initiation time point and forward, or retrospective when outcomes are collected from the past events/exposures that subjects already experienced. The focus of this editorial is randomized and controlled-clinical trials.

The design of a clinical trial must be developed in such way that objectives can be met and specific research questions answered. Many aspects should be taken into account while designing a clinical trial. Specific factors must be considered such as: utilization of control groups, randomization techniques, treatment assignments, allocations and blinding procedures (i.e., whether or not trial should be blinded to investigators and/or study subjects). Randomized, double-blind and placebo-controlled trials are considered the ‘gold standard’ of clinical trial design. Although such study design may not be appropriate or practical for some studies, as ‘one size’ doesn't fit all and an individual approach to each study design must be considered and evaluated prior to implementation.

## Utilization of control

Investigators can create more homogeneous groups for comparison when assignment to the study groups is performed in a controlled fashion. Therefore, investigators can conclude that differences observed in the groups are related to the study intervention, rather than attributed to the difference within the group of subjects. Examples of controls include utilization of the following: placebo (inert substance), active medication as comparator which was prior approved by regulatory agencies, currently approved device as comparator, a standard care medication/treatment or administration of different dose of the same medication (i.e., in dose-ranging studies) [1,2].

### Placebo control

According to Declaration of Helsinki, the use of placebo is acceptable in clinical trials where no standard treatment currently exists and there is a clinical equipoise, which means there is no consensus among healthcare experts regarding the best treatment for disease/condition of interest [3]. Subjects randomly assigned to active treatment group may or may not receive benefit from investigational product, but there will be a possibility that subjects assigned to placebo will avoid side effects or toxicities of investigational product with uncertain safety and efficacy profile [2,4]. When approved by regulatory agency, treatment already exists as part of routine clinical care and clinical equipoise does not, an investigational product should be compared with the standard, routinely utilized in clinical care treatment for that disease/condition rather than placebo, as withholding an effective and safe existing treatment is considered unethical in these circumstances [2,4].

### Sham procedures

In some device trials, subjects are randomized to a ‘sham procedure’ to minimize their bias, especially when assessing subjective end point such as self-reported by subjects’ symptoms (i.e., pain, discomfort, quality of life outcomes, etc.) [1,2]. For instance, in surgical trials, sham procedures can be used to mimic investigational device implantation to make experience in a control group as similar as possible to experimental one [1,2]. Under local anesthesia, subjects in control group will have a small incision made but the experimental device would not be actually implanted. In order to maintain the blind for subjects, the surgeon will verbally request the same surgical instruments and manipulate the part of body of the subject as they would have, if performing the real procedure in experimental group. In both the groups, the same outcomes will be assessed as indicated in study protocol [1,2].

### Randomization

Randomization was first introduced by the statistician and geneticist Sir RA Fisher, who used this technique in agricultural studies in Britain in the 1920s [5]. When first used in human subjects in early 1930s, randomization was literally accomplished by the toss of a coin to determine treatment assignment group. Currently, methods of randomization are much more sophisticated and diverse based on study design. They can include simple randomization, when research subjects are assigned to treatment group based on a code, which is generated by order of admission into the study; block randomization, where subjects are proportionally assigned to treatment groups within prespecified number or ‘block’ of subjects (i.e., all subjects from one floor in the hospital are assigned to one block and subject from a different floor to another); and stratification, when subjects are assigned to treatment subgroups based on demographics such as gender, age, weight, severity of the disease or any other relevant variables that can affect outcome [6,7]. Permuted block randomization adds an additional blinding element by varying the number of subjects in the block. Prior consideration must be given to the following: block size, number of varying block and avoidance of block sizes being multiplication of each other based on number of subjects required, trial design and the extent of stratification to ensure treatment balance. Random permuted blocks can cause treatment imbalances when trial stops mid-block [7]. Statistical methods can be applied as restrictions to the random permuted-block randomization study designs to mitigate occurrence of imbalances, while still achieving unbiased assessment of treatment differences. The smaller the block size, the more likely it is that balance is forced. As an example, in an unblended, open-label study randomizing 110 subjects with blocks of two subjects, it is easy to know every second participant's assignment in advance [6,7]. This would lead to a high probability of selection bias. The block size should not be known to anyone other than the unblinded statistician and it should be kept confidential until the study is completed to avoid bias.

A randomized controlled trial uses randomization to minimize bias in assigning subjects to different study groups. While some subjects are randomly assigned to receive the investigational product, others in the control group are assigned to standard of care treatment or placebo.

The goal of randomization is to have groups on the study as similar as possible, so that differences observed in subjects’ outcomes can be claimed to be caused by effect of the experimental intervention and not attributed to any other factors, which are typically referred to as confounding factors. Randomization takes care of balancing both known and unknown confounding factors, since subjects are randomly assigned to treatment groups [6]. Since none of the study groups are known to be superior at the beginning of the study (i.e., safety and efficacy of study drug/device is not proven yet vs control/or comparison treatment group), randomization is an ethical method of randomly assigning subjects to study groups as long as equipoise still exists. Despite of term ‘random’, the randomization scheme is typically developed by the statistician to address all relevant aspects of study design. Also statisticians can avoid imbalances in study groups by using a mode of randomization called dynamic allocation or a minimization algorithm [6,7]. Using this approach, if there have been a number of randomizations that have all been to the same arm/group of the study consecutively, the ‘weighting’ can be applied so that chances of getting the other result will be not 1:1, but some others such as 2:1, or 3:1. This ‘weighting’ can be held up until the study arms become balanced [6,7]. Other methods to prevent randomization imbalances can be used as well, such as block randomizations.

### Methods of randomization

Methods of randomization keep changing over time as new technologies evolve. Early randomizations were performed by opening sealed envelopes at each participating clinical research site. This method was likely to have some issues due to misplaced envelopes, some of them could have been opened out of order, somebody at the site could accidentally or intentionally tamper with the envelops. Currently, centralized randomization is the most common method utilized in clinical trials and it can be implemented either by phone or via internet portal. The phone can be utilized to access interactive voice response system, in which the caller, typically research nurse, coordinator or physician investigator follows instructions to activate randomization for a particular subject. Interactive voice response system is a very commonly used option because it provides quick access to randomization mechanism, as well as automated cross check and recording of eligibility criteria of the subjects prior to randomization into the study. Internet-based randomization platforms are becoming more often used, however, they depend on internet access at a particular site, which can be an issue in some remote locations with limited or an unstable internet access.

## Conclusion & future perspective

There are several advantages to randomization, such as that it ensures against the accidental bias in the experiment and produces groups similar in all aspects, except the intervention each group received. Also it produces comparable groups, so that validity of statistical tests of significance can be guaranteed.

Utilization of randomization was effectively demonstrated to the benefit of research in multiple studies. Simple randomization works well for the large clinical trials (n > 100) and for small to moderate clinical trials (n < 100) without covariates, use of block randomization helps to achieve the balance. For small to moderate size clinical trials with several prognostic factors or covariates, the recommendation is to utilize adaptive randomization method, which could be more useful in providing a means to achieve treatment balance.

Despite of currently being recognized as a ‘gold standard’ to conduct clinical trials, randomized-controlled clinical trials have some disadvantages. Participants studied may not represent general study population and it will affect generalizability of results. Recruitment can be hard and sometimes patient population is too small, if eligibility criteria were made too stringent or there are competing clinical trials recruiting participants at the same time. Randomization procedures can be administratively complex to carry over. In addition, acceptability of randomization process can vary between physicians and/or participants who will refuse to be assigned randomly to a study group based on their own perceptions (i.e., potential research subjects may think that randomization will delay their access to effective treatment which is already been approved, and a lot of them do not want to be assign to the placebo or cautious regarding unknown side effect of experimental product). Physician investigators may not be willing to randomly assign subjects based on their clinical characteristics and not have control over their course of treatment, especially in complex patients with advanced disease stage and unknown efficacy and/or safety profile of investigational product.

As of today, randomized controlled clinical trials remain the ‘gold standard’ to conduct prospective, interventional, clinical investigations with different randomization methods and types. They develop to produce comparable study groups and guarantee validity and statistical significance of results produced. Although ‘one size may not fit all’ but there are variety of techniques currently available to achieve this goal and improve clinical trial outcomes.
